# Audit of routine immunohistochemistry testing for mismatch repair proteins at diagnosis of colorectal cancer under the age of 50

**DOI:** 10.1186/1897-4287-10-S2-A78

**Published:** 2012-04-12

**Authors:** L Lipton, M Kentwell, M Li, D Williams, M Christie, A Landgren, C Dow, I Jones, S McLaughlin, M Delatycki, F Macrae, E Lynch

**Affiliations:** 1 Familial Cancer Centre, Melbourne Health, Royal Melbourne Hospital, Australia; 2 Clinical Genetics, Austin Health, Heidelberg Repatriation Hospital, Australia; 3 Department of Pathology, Melbourne Health, Royal Melbourne Hospital, Australia; 4 Department of Pathology, Austin Health, Heidelberg Repatriation Hospital, Australia; 5 Department of Pathology, Western Health, Western Hospital, Australia; 6 Department of Surgery, Melbourne Health, Royal Melbourne Hospital, Australia; 7 Department of Surgery, Western Health, Western Hospital, Australia; 8 Department of Colorectal Medicine and Genetics, Melbourne Health, Royal Melbourne Hospital, Australia

## Background

In May 2007, the Victorian Cancer Oncology Hereditary Bowel Cancer Group (VCOG HBCG) released a position statement in regards to the identification of Hereditary Non-Polyposis Colorectal Cancer (HNPCC) by immunohistochemistry (IHC) testing. This was based on the consensus among clinical groups, that most families with HNPCC are not being identified and strategies to improve identification should be implemented. The VCOG HBCG recommendations was to test all colorectal cancers in patients under 50 years of age by IHC for MLH1, MSH2, MSH6 and PMS2 proteins, as part of the routine pathological assessment of cancers presenting in these patients, without direct consent. This recommendation was supported by the Australian College of Pathologists and widely circulated to the clinical community from 2007.

The primary purpose of this audit was to ascertain the frequency of IHC being performed for consecutive patients diagnosed with colorectal cancer under 50 years of age, at three Victorian hospitals since the publication of the VCOG HBCG position statement. The purpose of this audit was also to ascertain the number of cases where IHC results showed loss of expression, which were referred to the Familial Cancer Centre (FCC) for further assessment and the outcome of this assessment.

## Methods

Lists of patients with colorectal cancer diagnosed under 50 years of age for the calendar years of 2007, 2008, 2009 and 2010 were extracted from each hospital database. Pathology reports for all patients were manually checked to assess cancer site, stage, mucinous component, tumour infiltrating lymphocytes and whether IHC was performed. Those patients with IHC absent results were cross-checked with FCC databases to see whether they were referred for further evaluation and what the outcome of attendance had been.

## Results

Data from three hospitals is presented. 204 patients were diagnosed with CRC under 50 years of age from Jan 2007 to Aug 2010. 164 had IHC testing as part of routine management: 67.5% (27/40) in 2007, 69.8% (44/63) in 2008, 66.6% (36/54) in 2009 and 72% (36/50) in 2010. One hospital showed a greater increment in rate of IHC uptake over time. This hospital changed its synoptic pathology report to include IHC.

## Conclusion

There were differences between hospitals in the uptake of the recommendation to implement routine IHC for patients diagnosed with colorectal cancer under 50 years of age. Barriers and enablers were identified for initiating routine IHC testing at the hospitals. These include addressing concerns about consent and onus of consent acquisition if needed, confusion about whether the IHC testing is a genotype or phenotype test, translation of the findings to clinical practice, establishment and optimization of the IHC testing, and funding of IHC testing.

